# Ankle impingement

**DOI:** 10.1186/s13018-016-0430-x

**Published:** 2016-09-09

**Authors:** Kyle P. Lavery, Kevin J. McHale, William H. Rossy, George Theodore

**Affiliations:** Division of Sports Medicine, Department of Orthopaedic Surgery, Massachusetts General Hospital, Harvard Medical School, 175 Cambridge Street, Suite 400, Boston, MA 02114 USA

**Keywords:** Ankle impingement, Ankle arthroscopy, Os trigonum

## Abstract

Ankle impingement is a syndrome that encompasses a wide range of anterior and posterior joint pathology involving both osseous and soft tissue abnormalities. In this review, the etiology, pathoanatomy, diagnostic workup, and treatment options for both anterior and posterior ankle impingement syndromes are discussed.

## Background

Impingement refers to abnormal entrapment or contact of structures resulting in pain or restricted motion. Impingement syndromes are a commonly recognized source of musculoskeletal symptoms in many areas, notably subacromial impingement in the shoulder and femoroacetabular impingement in the hip. Similarly, impingement syndromes are an increasingly recognized source of pain and disability in the ankle.

Impingement syndromes in the ankle include a broad spectrum of pathology with varying etiologies, anatomic features, and presentations. Although no official classification exists, these syndromes are generally defined by the particular anatomic area involved. Specific anterior, anterolateral, anteromedial, posterior, posteromedial, posterolateral, and syndesmotic impingements have been described [1, 2]. However, these pathologies are generally grouped into anterior and posterior impingement syndromes for simplicity.

Anterior ankle impingement syndrome results from compression of structures at the anterior margin of the tibiotalar joint during dorsiflexion. Anterior impingement has long been recognized as a cause of pain in athletes. In 1949, McMurray described “footballer’s ankle”, a commonly observed condition in professional soccer players involving anterior osteophytes of the dorsal talar neck and distal tibia. The term was later refined to “impingement exostoses” by O’Donoghue in 1957 to include other patient populations [3].

Likened to a “nut in a nutcracker”, posterior impingement syndrome is characterized by compression in the anatomic region between the posterior tibia and calcaneus during plantar flexion. While anatomists and surgeons have long recognized structures at risk for compression, such as the os trigonum, the operative treatment of posterior impingement was not reported until 1982 when Howse described treating a “posterior block of the ankle joint” in a population of elite dancers [4]. The presentation was later termed “talar compression syndrome” [5].

Ankle impingement is an increasingly recognized cause of symptoms in an athletic population as our understanding of the etiology, pathogenesis, and presentation continues to evolve. Recent advancements in diagnostic and treatment techniques aim to improve outcomes.

## Main text

### Etiology and pathoanatomy

#### Anterior impingement

Anterior ankle impingement generally refers to entrapment of structures along the anterior margin of the tibiotalar joint in terminal dorsiflexion. Multiple osseous and soft tissue anatomic abnormalities have been recognized as causative factors.

Characteristic spurs or “exostoses” at the anterior distal tibia and dorsal talar neck have long been observed in athletes with anterior ankle pain and limited motion. Isolated talofibular lesions have also been described [6]. The morphology of anterior tibiotalar exostoses has been well-studied, and cadaveric dissections have found these lesions to be intra-articular, well within the distal tibial and dorsal talar capsular attachments [7, 8].

Although they are often referred to as “kissing osteophytes”, these tibial and talar spurs surprisingly often do not actually overlap and abut. Evaluation of preoperative CT scans has shown that talar spurs generally lie medial to the midline of the talar dome and tibial spurs are generally located laterally [9]. A distinct trough in the articular talar dome often “accepts” the tibial osteophyte during dorsiflexion. Kim, et al. referred to this as a “tram-track lesion” [10], while Raikin, et al. termed it a “divot sign” [11]. Subsequent studies have confirmed a high rate of corresponding talar cartilage lesions (80.7 %) and loose bodies in patients with distal tibial lesions [12].

Anterior intra-articular soft tissues may contribute to impingement in isolation or in conjunction with bony lesions. A triangular soft tissue mass composed primarily of adipose and synovial tissues exists in the anterior joint space. These tissues are compressed after 15° of dorsiflexion in asymptomatic individuals [7]. Anterior osteophytes may limit the space available for this soft tissue and exacerbate its entrapment, resulting in chronic inflammation, synovitis, and capsuloligamentous hypertrophy. Post-traumatic fibrous bands [13], thickened anterior tibiofibular ligaments [14, 15], and synovial plica [16], have also been identified as causative factors.

While the impinging anatomic lesions have been well described, their exact etiology is less well understood. Early reports hypothesized spurs to be enthesophytes caused by traction to the anterior capsule during repetitive plantar flexion [3]. However, anatomic studies have demonstrated the chondral margins and lesions to be deep to the joint capsule rather than at its attachment, resulting in the traction, disproving the traction theory [7–9]. More recent observations of athletic populations commonly affected by anterior impingement have led to hypotheses that pathology occurs due to repetitive impaction injury to the anterior chondral margin from hyper-dorsiflexion or direct impact from an external object such as a soccer ball [17, 18].

Chronic lateral ankle instability has also been hypothesized to contribute to the development of both bony and soft tissue lesions associated with anterior impingement due to abnormal repetitive micromotion [14, 19]. Multiple studies have examined the prevalence of associated anterior impingement lesions at the time of arthroscopy in patients undergoing stabilization procedures for lateral ankle instability. Soft tissue lesions, such as synovitis in the anterior compartment or anterior lateral gutter, have been observed with high frequency (63–100 %), while anterior tibial osteophytes have often been found consistently (12–26.4 %) [20–22]. In one study, patients undergoing a Brostrom procedure had 3.37 times the incidence of bone spurs than matched asymptomatic controls [23].

#### Posterior impingement

Posterior ankle impingement results from compression of structures posterior to the tibiotalar and talocalcaneal articulations during terminal plantar flexion. Similarly, this can be caused by multiple osseous and soft tissue etiologies in isolation or in combination.

**Fig. 1:**
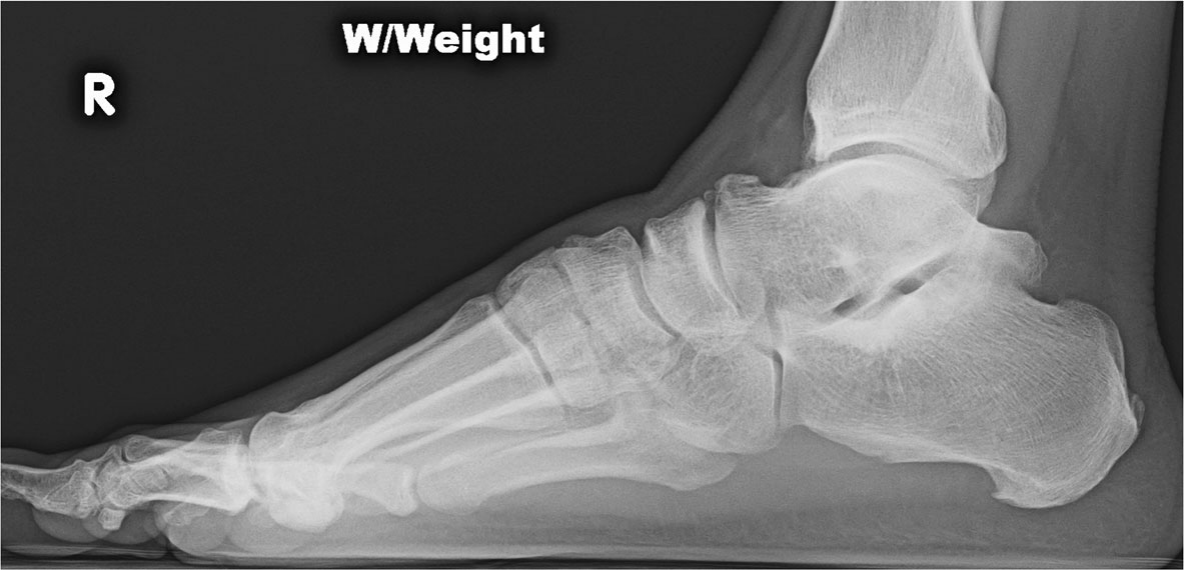
A lateral radiograph demonstrates an elongated posterolateral (trigonal) process of the talus (Stieda process)

**Fig. 2:**
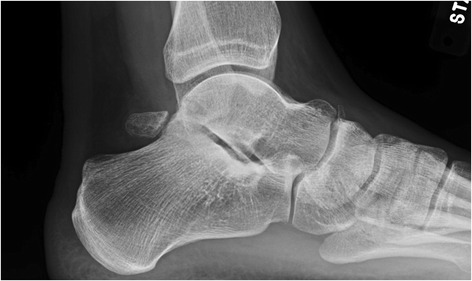
A lateral radiograph demonstrates a large os trigonum

Pathology associated with the lateral (trigonal) process of the posterior talus is the most common cause of posterior impingement (Fig. 1). Anatomic variants of this structure have been well described. A Stieda process refers to an elongated tubercle. An os trigonum may represent failure of fusion of a secondary ossification center to the talar body, although this structure has been heavily debated in the orthopedic and radiologic literature. Impingement related to the trigonal process can result from acute fracture, chronic injury due to repetitive microtrauma, or mechanical irritation of the surrounding soft tissues [24] (Fig. 2).

Less commonly, posterior symptoms may result from tibiotalar or subtalar degenerative joint disease due to osteophyte impingement or associated reactive hypertrophic capsule and synovium. Post-traumatic sequelae from fracture malunion of the posterior malleolus, talus, or calcaneus may also occur [25]. A case of talar osteonecrosis resulting in posterior impingement has also been reported [26].

Various soft tissue structures may cause posterior impingement symptoms as well. Posterior capsuloligamentous injury due to repetitive or acute hyperflexion can lead to inflammation, scarring and thickening of the capsule, posterior inferior tibiofibular ligament, and posterior fibers of the deltoid ligament [27–29] (Fig. 5). The flexor hallucis longus (FHL) tendon, running between the medial and lateral posterior processes of the talus, is commonly affected by tenosynovitis and tendinosis. The tendinopathy may result from overuse or secondary to irritation from surrounding abnormal bony anatomy. Anatomic soft tissue variants, such as the posterior intermalleolar ligament and several anomalous muscles, have been described as other sources of impingement [30–34].

### Presentation

#### Anterior impingement

Anterior impingement syndrome typically presents as anterior ankle pain during terminal dorsiflexion. Exacerbating activities commonly include climbing stairs, running or walking up hills, ascending ladders, and deep squatting. The classic association with competitive soccer players has long been recognized, but the reason that this subset of athletes is commonly affected is unclear [10, 17, 18]. In the later stages, dorsiflexion may be limited secondary to mechanical block or pain, creating a cycle of progressive joint stiffness and loss of function. In isolated soft tissue lesions, the patient may report a subjective popping or snapping sensation.

#### Posterior impingement

Posterior impingement syndrome generally presents as a less specific pain deep to the Achilles tendon and may often be confused with Achilles or peroneal tendon pathology. Symptoms may be worsened by activities involving plantar flexion and repetitive push-off maneuvers, including downhill running and walking, descending stairs, and high-heeled shoe wear. Posterior impingement classically presents in dancers, specifically those participating in classic ballet, presumably due to repetitive weight bearing in the plantar-flexed “en-pointe” and “demi-pointe” positions [35–38]. In a recent systematic review, dancers represented 61 % of patients undergoing surgery for posterior impingement [39]. It has also been reported to affect fast-bowlers in cricket [40].

### Physical examination

A comprehensive physical examination of the foot and ankle should be performed when assessing for impingement syndromes. The ankle and foot are inspected for abnormal alignment, joint effusion, or soft tissue edema. The bone and soft tissue structures are systematically palpated to assess for localized tenderness. While anterior or anterolateral tenderness is characteristic in anterior impingement, posterior impingement signs can be more difficult to elicit and localize, as structures are deeper. Posteromedial ankle tenderness with resisted plantar flexion of the first metatarsophalangeal joint is more consistent with FHL pathology, while posterolateral tenderness with forced ankle plantar flexion is more likely to involve pathology associated with the trigonal process.

Passive and active ranges of motion of the joints bilaterally are measured, including dorsiflexion, plantar flexion, subtalar, and midfoot motions. Laterally, the peroneal tendon is assessed for tenderness, deformity, or subluxation. The sural nerve is evaluated for sensitivity. Posteriorly, the Achilles tendon is assessed for fusiform enlargement or retrocalcaneal bursitis. Medially, the tibial nerve is evaluated for tarsal tunnel syndrome, and the posterior tibial tendon’s function is assessed. The anterior drawer and talar tilt tests of the tibiotalar joint are performed to exclude ankle instability. Finally, a straight leg raise test in the seated or supine position may be done to exclude an L5 or S1 radiculopathy.

### Imaging

Imaging of an ankle suspected of impingement should begin with a plain x-ray series, as the diagnosis is often confirmed with simple radiographs. Initial views should include weight-bearing AP, lateral, and mortise projections. Careful attention is given to the lateral view, assessing for exostoses on distal anterior tibia and dorsal talar neck and posterior bony abnormalities, including a Stieda process or os trigonum.

Alternative oblique views have been described for both anterior and posterior impingement lesions to better assess for bony abnormalities, as standard views can miss some lesions. To detect anteromedial lesions, the beam is aimed 45° craniocaudad with the leg externally rotated 30° [41]. The utility of the oblique anteromedial impingement view has been confirmed to have a higher sensitivity in detecting both tibial (85 vs. 40 %) and talar (73 vs. 32 %) osteophytes when added to a standard lateral radiograph [42]. Lesions associated with the trigonal process are best viewed on a 25° external rotation-lateral view [43]. Dynamic hyper-plantar-flexed or dorsiflexed laterals can be considered to demonstrate abnormal bony contact.

**Fig. 3:**
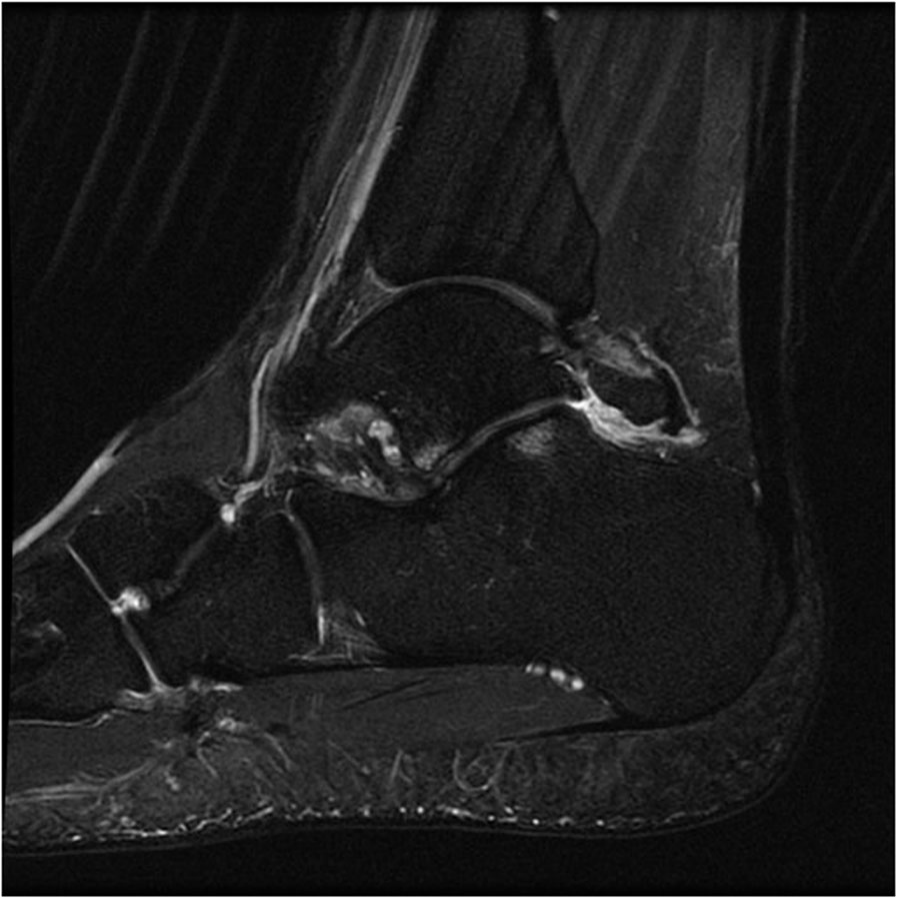
A T2-weighted MRI with reactive edema surrounding an os trigonum

Advanced imaging, such as MRI, may also be considered when the diagnosis remains inconclusive. Images should be evaluated for bone edema, effusion, synovitis, tenosynovitis, and concomitant chondral injury (Fig. 3). In anterior soft tissue impingement, the anterolateral gutter may contain hypertrophic synovium or fibrosis. Increased marrow signal intensity at the trigonal process or os trigonum is suggestive of an acute injury or chronic stress fracture [44]. The efficacy of MRI in evaluating soft tissue impingement lesions is variable, with reported sensitivities 42–89 % and specificities 75–100 % [45–50]. Computed tomography has been used in defining the morphology of bony lesions for planning surgical resections [51]. Recently, ultrasound has also gained popularity as a reliable and inexpensive modality in evaluating impingement lesions and administering therapeutic injections [52, 53].

### Nonsurgical treatment

Nonsurgical treatment remains the initial approach to the management of both anterior and posterior impingement syndromes, despite limited evidence of its efficacy. For acute symptoms, a period of rest and an avoidance of provocative activities are recommended. This approach can be supplemented with ice, NSAIDs, or cast immobilization in more severe cases. Rest can be supplemented with ice, NSAIDs, or immobilization in severe cases. In chronic cases, shoe modifications, including heel lift orthoses to prevent dorsiflexion, have been utilized. Physical therapy protocols focus on improving ankle stability and optimizing proprioception. Authors have reported successful symptom relief with ultrasound-guided corticosteroid injections, which may also have diagnostic uses [54, 55].

### Surgical treatment

Surgical intervention is generally indicated for persistent symptoms which have not responded to non-operative treatment, affected normal activities of daily living or athletic performance, and correlated with physical exam and imaging findings. The surgical approach and technique vary by the anatomic region and pathology involved.

#### Anterior impingement

Surgical goals for the treatment of anterior impingement involve removing the offending pathologic lesion contributing to the symptoms. This may involve resection or debridement of bony lesions, soft tissue lesions, or both. Early studies described the use of open anterior or lateral arthrotomy [3]. A lateral arthrotomy is often still utilized if a lateral ligamentous procedure is being performed concurrently. However, open approaches have largely been replaced by arthroscopic techniques [56–80].

**Fig. 4:**
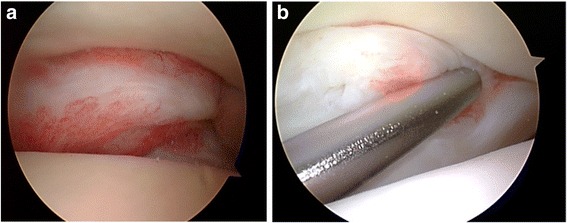
**a** An arthroscopic image demonstrates anterolateral scar impingement with associated synovitis. **b** An arthroscopic shaver is used to resect the lesion

Hawkins is credited with reporting the first arthroscopic approach for the treatment of bony anterior ankle impingement in 1988, citing improved visualization with a less invasive approach [81]. Standard anterolateral and anteromedial portals are typically utilized and may be extended with conversion to open arthrotomy if necessary. An arthroscopic burr is used to reshape the anterior tibia and dorsal talus to their native contours. A combination of a shaver and electrothermal device is used to debride hypertrophic or inflamed synovium and fibrotic tissue (Fig. 4). Intraoperative fluoroscopy may be used to confirm adequate resection of spurs (Fig. 4).

**Fig. 5:**
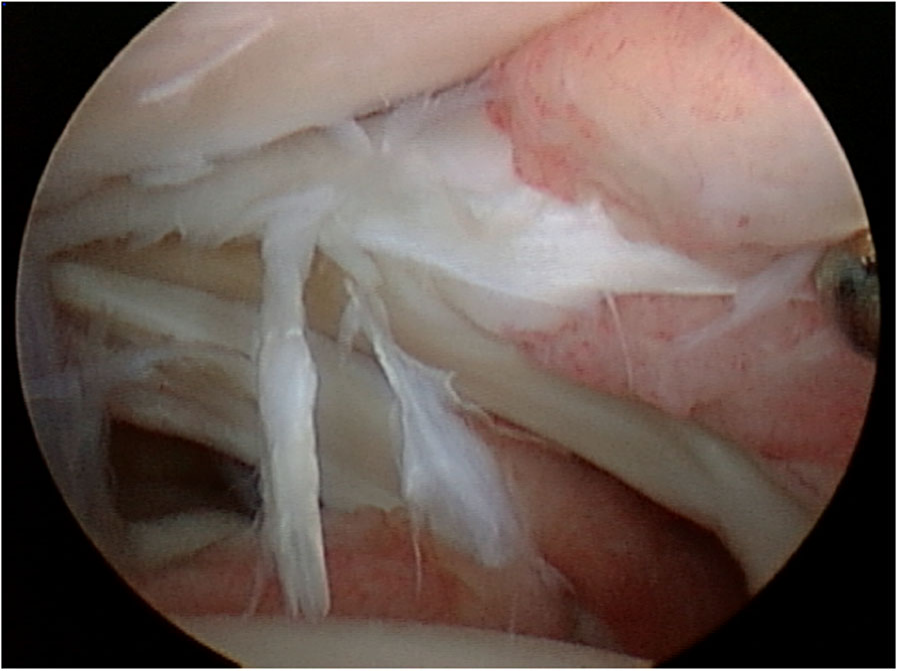
An arthroscopic image demonstrates tearing of the posterior inferior tibiofibular ligament complex

Zwiers et al. conducted a recent systematic review examining the results of the arthroscopic treatment of anterior impingement [75] (Fig. 5). The review included 19 studies and 905 patients, with an average age of 32.7 years. At a combined mean follow-up 35.3 months, 74–100 % of patients were satisfied with the results of their procedure. AOFAS scores improved consistently, ranging from 34–75 preoperatively and increasing to 83.5–92 postoperatively. There was a 5.1 % overall complication rate, with 1.2 % considered major complications. This is consistent with a 4 % complication rate in a previous review by Simonson et al. [82].

#### Posterior impingement

Similarly, the surgical goal of treating posterior impingement involves resection of the causative anatomy. Most commonly, symptom relief is achieved by excision of a painful trigonal process or os trigonum, with debridement of surrounding inflammatory or hypertrophic soft tissues.

Posterior pathology can be targeted through an open lateral, open medial, or endoscopic approach. A lateral approach allows for more direct access to the trigonal process with less risk to the medial neurovascular bundle. A medial approach allows for concomitant FHL pathology to be addressed more easily. Since 2000, posterior endoscopic approaches have gained popularity, with the potential for faster return to sport and lower complication rates [31, 83–97]. With the patient positioned prone, posteromedial and posterolateral hindfoot portals adjacent to the Achilles tendon typically provide excellent access to extra-articular posterior structures.

Ribbans et al. reviewed 47 papers consisting of 905 patients treated surgically with both open and endoscopic approaches for posterior impingement [39]. Eighty-one percent of symptoms were attributed to osseous pathology. In the included series, 67–100 % of patients experienced good or excellent outcomes. Zwiers et al. conducted a similar systematic review including 16 studies [98]. Significantly lower complication rates (7.2 vs. 15.9 %) and earlier return to full activity (11.3 vs. 16 weeks) were found with endoscopic surgery.

## Conclusions

Ankle impingement can encompass a broad spectrum of anterior and posterior pathology involving both osseous and soft tissue abnormalities. While anterior impingement produces symptoms with terminal dorsiflexion, posterior impingement is exacerbated by activities involving hyper-plantar flexion. History, physical examination, imaging studies, and diagnostic injections all contribute to the accurate diagnosis of the condition. Many patients will respond favorably to non-operative treatment modalities, but both open and arthroscopic techniques have evolved to address chronic problems with successful and predictable outcomes.
